# Steady‐state chemoreflex drive captures ventilatory acclimatization during incremental ascent to high altitude: Effect of acetazolamide

**DOI:** 10.14814/phy2.15521

**Published:** 2022-12-02

**Authors:** Valerie C. Cates, Christina D. Bruce, Anthony L. Marullo, Rodion Isakovich, Gurkarn Saran, Jack K. Leacy, Ken D. O′Halloran, Thomas D. Brutsaert, Mingma T. Sherpa, Trevor A. Day

**Affiliations:** ^1^ Department of Biology, Faculty of Science and Technology Mount Royal University Calgary Alberta Canada; ^2^ Department of Physiology. School of Medicine University Cork College Cork Ireland; ^3^ Department of Exercise Science Syracuse University Syracuse New York USA; ^4^ Kunde Hospital Khunde Nepal

**Keywords:** acetazolamide, acid–base, carbonic anhydrase inhibitor, high altitude, hypoxic ventilatory response, novel methodology, renal compensation, respiratory chemoreflexes, steady‐state chemoreflex drive, ventilatory acclimatization

## Abstract

Ventilatory acclimatization (VA) is important to maintain adequate oxygenation with ascent to high altitude (HA). Transient hypoxic ventilatory response tests lack feasibility and fail to capture the integrated steady‐state responses to chronic hypoxic exposure in HA fieldwork. We recently characterized a novel index of steady‐state respiratory chemoreflex drive (SSCD), accounting for integrated contributions from central and peripheral respiratory chemoreceptors during steady‐state breathing at prevailing chemostimuli. Acetazolamide is often utilized during ascent for prevention or treatment of altitude‐related illnesses, eliciting metabolic acidosis and stimulating respiratory chemoreceptors. To determine if SSCD reflects VA during ascent to HA, we characterized SSCD in 25 lowlanders during incremental ascent to 4240 m over 7 days. We subsequently compared two separate subgroups: no acetazolamide (NAz; *n* = 14) and those taking an oral prophylactic dose of acetazolamide (Az; 125 mg BID; *n* = 11). At 1130/1400 m (day zero) and 4240 m (day seven), steady‐state measurements of resting ventilation (V̇_I_; L/min), pressure of end‐tidal (P_ET_)CO_2_ (Torr), and peripheral oxygen saturation (SpO_2_; %) were measured. A stimulus index (SI; P_ET_CO_2_/SpO_2_) was calculated, and SSCD was calculated by indexing V̇_I_ against SI. We found that (a) both V̇_I_ and SSCD increased with ascent to 4240 m (day seven; V̇_I_: +39%, *p* < 0.0001, Hedges' *g* = 1.52; SSCD: +56.%, *p* < 0.0001, Hedges' *g* = 1.65), (b) and these responses were larger in the Az versus NAz subgroup (V̇_I_: *p* = 0.02, Hedges' *g* = 1.04; SSCD: *p* = 0.02, Hedges' *g* = 1.05). The SSCD metric may have utility in assessing VA during prolonged stays at altitude, providing a feasible alternative to transient chemoreflex tests.

## Graphical abstract

During incremental ascent to 4240 m over seven days, both ventilation and a novel metric of chemoreflex drive (SSCD) were augmented, which further increased in a subgroup taking an oral prophylactic dose of acetazolamide (Az), compared to those that were Az free. The novel SSCD metric may have utility in assessing ventilatory acclimatization during incremental high altitude ascent.

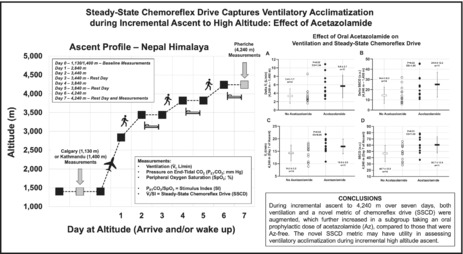

## 1 INTRODUCTION

Ventilatory acclimatization (VA) to high altitude (HA), arguably one of the most important responses to HA ascent (Ainslie et al., 2013; West, 2006), is mediated through changes in both peripheral and central chemoreceptor sensitization and/or central integration (Moya et al., 2020; Robbins, 2007). In response to chronic HA exposure, the sensitivity of the peripheral chemoreflex is augmented driving (a) increases in the magnitude of the hypoxic ventilatory response (HVR; Howard & Robbins, 1995; Sato et al., 1992; Teppema & Dahan, 2010; White et al., 1987), and subsequently (b) increases steady‐state breathing while hypoxic (Duffin & Mahamed, 2003; Eger et al., 1968; Michel & Milledge, 1963). The central chemoreflex is also augmented during chronic exposure to hypoxia (Fan et al., 2010), in part due to the renally mediated elimination of bicarbonate ions (HCO_3_^−^) and associated reduced buffering capacity in the central compartment, as the kidneys compensate for the sustained respiratory alkalosis (Krapf et al., 1991; Mathew et al., 1983; Pitts et al., 1948; Schoene et al., 1990; Severinghaus et al., 1963). This renal elimination of HCO_3_^−^ in the context of chronic hypobaric hypoxia increases the relative stimulation of central chemoreceptors via [H^+^] for a given CO_2_ challenge (e.g., Ainslie et al., 2013; Fan et al., 2010). Thus, these respiratory and acid–base perspectives are relevant to the integrative understanding of the chemoreflex control of breathing and VA to HA.

There are a number of methods used to assess HVR magnitude in a laboratory context (Teppema & Dahan, 2010), including rebreathing (e.g., Duffin, 2011), dynamic end‐tidal forcing (e.g., Steinback & Poulin, 2007), and transient gas‐perturbation tests (e.g., Pfoh et al., 2016). Unfortunately, consensus has not yet been reached regarding standard accepted methodology (e.g., Powell, 2006), and differential methods and/or contexts complicate the comparison between methods at sea level and/or between sea level versus HA (e.g., Pfoh et al., 2016, 2017; Steinback & Poulin, 2007; Teppema & Dahan, 2010). Despite the importance of assessing VA with ascent, transient laboratory tests of the central or peripheral chemoreflexes in humans lack portability and feasibility in HA fieldwork. In addition, HVR testing in the context of HA raises technical and theoretical challenges, where (a) participants are already hypoxemic, raising safety concerns, (b) participants are hypocapnic at baseline compared to sea level, affecting both central and peripheral chemoreflex activation or sensitivity, and (c) acid–base status may be altered, with renal compensation (i.e., HCO_3_^−^ elimination) occurring at variable magnitudes and rates with ascent profiles (c.f., Bird, Leacy, et al., 2021; Zouboules et al., 2018). Most importantly, ventilatory responses to transient hypoxic gas challenges do not reflect the steady‐state ventilatory strategy employed while at rest in sustained hypobaric hypoxic contexts. There is a need for alternative, integrative, and portable methods to assess chemoreflex drive in the context of VA during HA ascent.

We recently proposed and characterized a novel index of steady‐state chemoreflex drive (SSCD) as an alternative method to assessing VA during ascent to HA (Bruce et al., 2018; Leacy et al., 2021; Pfoh et al., 2017; see methods for details). The SSCD metric characterizes the ventilatory strategy employed in relation to prevailing chemostimuli in the steady‐state. First, a “stimulus index” (SI) is calculated (P_ET_CO_2_/SpO_2_), given ventilation is known to be linearly and directly proportional to changes in P_ET_CO_2_ (e.g., Duffin, 2011; Nielsen & Smith, 1952) and linearly and inversely proportional to changes in SpO_2_ (e.g., Rebuck & Campbell, 1974). Second, ventilation is then indexed against the SI to calculate SSCD. We previously introduced the rationale and potential utility of the SSCD metric relative to more commonly utilized transient peak response HVR tests, in particular when assessing VA during incremental ascent to HA (Bruce et al., 2018; Pfoh et al., 2017). Preliminary data in a small cohort of trekkers demonstrated that the SSCD increased with incremental ascent to 5160 m, capturing VA in lowlanders (Bruce et al., 2018). We also recently found that ascent‐descent hysteresis in SSCD (to quantify VA magnitude) was associated with lower maximum acute mountain sickness (AMS) scores during incremental ascent to and descent from 5160 m (Leacy et al., 2021). However, the SSCD metric has not been characterized and compared between those taking a prophylactic dose of acetazolamide and those that were acetazolamide‐free.

Oral acetazolamide administration is commonly used to aid in VA, thus preventing or treating altitude‐related illnesses, including AMS (e.g., Luks et al., 2019; Swenson, 2016). AMS occurs in 10%–25% of unacclimatized individuals ascending to >2500 m and ~70% of those who ascend to >4500 m (Karinen et al., 2008; Vardy et al., 2006). Acetazolamide, a carbonic anhydrase inhibitor, elicits metabolic acidosis via actions in the renal tubules, leaving a portion of normally filtered HCO_3_^−^ to be excreted in the urine, and concomitant retention of H^+^ in blood and body fluids (Swenson, 2014). Acetazolamide‐induced metabolic acidosis aids in VA by stimulating both central and peripheral respiratory chemoreceptors, and thus increasing ventilation and subsequent oxygenation (Swenson, 2016).

Aside from oral acetazolamide administration to treat AMS at altitude (250 mg BID; 29), lower doses are routinely administered prophylactically to prevent AMS (e.g., 125 mg BID), particularly during rapid ascent and/or higher ascent profiles (Kayser et al., 2012; Luks et al., 2019; van Patot et al., 2008). Despite this, the lowest recommended acetazolamide dose that limits side effects and prevents AMS has been inconsistent, ranging from 750 mg/day (Dumont et al., 2000) to the current recommended 250 mg/day (Gao et al., 2021; Kayser et al., 2012; Low et al., 2012; Luks et al., 2019; Nieto Estrada et al., 2017). However, more recent reports indicate that an even lower dose of 62.5 mg BID can be as effective as the standard 125 mg BID (Lipman et al., 2020; McIntosh et al., 2019).

It has yet to be determined if SSCD captures VA in a large group of trekkers during incremental ascent to 4240 m, and the potential superimposed effects of a prophylactic oral acetazolamide dose (125 mg BID) have not been characterized. Therefore, we sought to characterize VA via ventilation, the SSCD metric, and self‐reported AMS scores, and compare an acetazolamide‐free group (no acetazolamide; NAz) to one taking an oral prophylactic dose of acetazolamide (Az; 125 mg BID) during incremental ascent to HA (4240 m over 7 days) in the Nepal Himalaya. We hypothesized that (a) ventilation and SSCD would increase with incremental ascent to altitude, tracking VA, and that (b) prophylactic oral acetazolamide administration would elicit larger increases in ventilation and SSCD and (c) the Az group would report lower AMS symptoms than the NAz group following 7 days of incremental ascent to 4240 m.

## 2 METHODS AND MATERIALS

### 2.1 Participant recruitment

The current study took place in the context of several research expeditions to high altitude in the Nepal Himalaya. Participants were recruited via verbal communication, and each provided verbal and written, informed consent to undergo repeated measurements before and during ascent prior to voluntary participation in the study. Participants were all non‐smokers and had no self‐reported history of neurological, cardiovascular, respiratory, or metabolic illnesses, or taking any related medications, aside from hormonal birth control (see section below regarding dosage of acetazolamide). Due to their participation in organized and/or guided expeditions with pre‐determined dates, ovarian cycle in female participants could not be a criterion for inclusion/exclusion in this study, nor was it tracked or controlled for. Furthermore, assessing potential sex differences were not planned a priori. However, previous reports have demonstrated that cycling ovarian hormones do not affect central or peripheral chemoreflex magnitude (e.g., Macnutt et al., 2012). All participants abstained from alcohol and exercise for at least 12 h prior to measurements, which were all obtained on rest days (i.e., no trekking) following one night at the measurement altitude. At no time was any participant included in the NAz group taking acetazolamide or corticosteroids for the prevention or treatment of altitude‐related illnesses.

For transparency, there is some overlap of data with previous publications from our group. Specifically, ancillary cardiorespiratory measures of some participants were included in a number of recent publications: (Bird, Kalker, et al., 2021) assessing central sleep apnea with ascent; (Bruce et al., 2018) initially characterizing SSCD with incremental ascent; (Holmström et al., 2021) assessing splenic function with ascent; (Lafave et al., 2019) assessing cerebral blood flow with ascent; (Leacy et al., 2021) assessing cardiorespiratory hysteresis with ascent and descent.; (Zouboules et al., 2018) assessing arterial acid–base variables with ascent. However, the characterization of ventilation and SSCD to assess VA and AMS with incremental ascent in a large cohort of trekkers to 4240 m with (a) a calibrated pneumotachometer to measure ventilation, (b) corrected P_ET_CO_2_ to known PaCO_2_ values during incremental ascent (see Section 2), (c) comparison between subgroups with and without prophylactic oral acetazolamide and (d) assessing its potential relationship of SSCD to self‐reported AMS scores, are all novel aspects reported within this comprehensive methodological characterization. Here, we assess the SSCD metric with potential utility in tracking VA with incremental ascent to 4240 m in a large group of trekkers and assess the effect of a prophylactic dose of oral acetazolamide during ascent. Further, here we also outline a number of methodological considerations for its utility and future application.

### 2.2 Experimental protocols

#### 2.2.1 Ascent profile

Participants were recruited from three large research expeditions to HA, where separate groups of participants completed an identical ascent profile from 1400 m to 4240 m over 6 days, with final measures made on a rest day following sleeping at 4240 m on day seven. Due to logistical constraints associated with large expeditions and participant recruitment, baseline measures were either performed in Calgary (1130 m) or 2–3 days after arrival in Kathmandu (1400 m). All participants then flew together as a group from 1400 m to 2840 m (Lukla airport) for the first trekking day to 2840 m (Monju). They arrived at 3440 m (Namche) on day two and spent a full rest day there, free of hiking or elevation gain (day three). They then ascended to either 3820 m (Deboche) or 3860 m (Tengboche) on day four and stayed for a rest day (day five). Finally, they ascended to 4240 m (Pheriche) on day six and stayed for a rest day (day seven), where the subsequent measures for this study took place.

The atmospheric pressure (P_ATM_) at each altitude was not measured directly but rather calculated using a standard equation.

(1) $$P_{\text{Altitude}}=P_{0}e^{\frac{-\mathrm{mgh}}{\mathrm{kT}}},$$

where P_0_ = 101,325 Pa (sea level pressure), m = 4.81 × 10^−26^ kg (mass of one air molecule), *g* = 9.81 m s^−2^ (acceleration due to gravity), h = altitude, k = Boltzmann's constant (1.3806 × 10^−23^ m^2^ kg s^−2^ K^−1^), and *T* = 288.16 K (standard temperature at sea level). *P*_Altitude_ was then converted from Pa to mmHg (mmHg = Pa × 0.00750062) and reported in Tables 1 and 2 to illustrate the relative reduction in P_ATM_ and P_I_O_2_ with ascent.

**TABLE 1:** Resting ancillary and cardiorespiratory variables before and after incremental ascent to high altitude, comparing low and high altitude. Values are reported for the combined participant group (*n* = 25) and each subgroup, both the no acetazolamide group (NAz; *n* = 14) and acetazolamide groups (Az; *n* = 11). P_ATM_, atmospheric pressure (calculated; see Section 2); P_I_O_2_, pressure of inspired oxygen ([P_ATM_‐47] × 0.21); HR, heart rate; MAP, mean arterial pressure; [Hb], hemoglobin concentration; CaO_2_, oxygen content; P_ET_CO_2_, pressure of end‐tidal CO_2_ (corrected, see Section 2); SpO_2_, peripheral arterial oxygen saturation; V̇_I_, minute ventilation; SSCD, steady‐state chemoreflex drive (V̇_I_/SI); see Section 2). MAP, P_ET_CO_2_, urine pH, and AMS scores from morning ancillary measures (06:00–08:00), after one night's sleep after arrival at each altitude (1400 and 4240 m). All other measures from LabChart data during rest day at each altitude (09:00–17:00). Statistical tests utilized were two‐tailed paired t‐tests comparing 1130/1400 m and 4240 m for each variable, with *p*‐values listed for each test. For AMS scores, non‐parametric Wilcoxon signed‐ranks tests were utilized. Asterisk (*) indicates a significant difference from 1130/1400 m (*p* < 0.05). Values are reported as mean ± standard deviation, except for AMS scores, which are reported as median (range)

Variable/location	1130/1400 m (Day 0)	4.240 m (Day 7)	*p*‐value
P_ATM_ (mmHg)	665/644	460	N/A
P_I_O_2_ (mmHg)	130/125	87	N/A
**Group**	**All (*n* = 25)**		
HR (min^−1^)	76.2 ± 10.1	75.5 ± 14.9	*p* = 0.77
MAP (mmHg)	93.1 ± 9.7	99.5 ± 8.7*	*p* < 0.0001
[Hb] (g/L)	139.2 ± 12.6	149.5 ± 12.5*	*p* = 0.004
CaO_2_ (ml/dl)	18.2 ± 1.7	17.7 ± 1.5	*p* = 0.12
P_ET_CO_2_ (mmHg)	32.0 ± 4.2	26.0 ± 2.6*	*p* < 0.0001
SpO_2_ (%)	96.5 ± 1.3	87.0 ± 2.5*	*p* < 0.0001
SI (a.u.)	0.33 ± 0.04	0.30 ± 0.03*	0.0006
V̇_I_ (L/min)	11.1 ± 2.4	15.5 ± 3.3*	*p* < 0.0001
SSCD (a.u.)	33.9 ± 7.7	52.9 ± 14.5*	*p* < 0.0001
AMS Score	0 (0–1)	0 (0–2)*	*p* < 0.05
**Group**	**No Acetazolamide Group (*n* = 14)**		
HR (min^−1^)	75.6 ± 11.6	73.4 ± 15.0	*p* = 0.4
MAP (mmHg)	97.4 ± 7.8	103.0 ± 7.2*	*p* = 0.003
[Hb] (g/L)	140.0 ± 14.6	148.8 ± 14.6	*p* = 0.1
CaO_2_ (ml/dl)	18.3 ± 2.0	17.4 ± 1.8	*p* = 0.15
P_ET_CO_2_ (mmHg)	33.0 ± 5.1	27.0 ± 2.9*	*p* = 0.0007
SpO_2_ (%)	96.2 ± 1.1	86.4 ± 2.2	*p* < 0.0001
SI (a.u.)	0.34 ± 0.05	0.31 ± 0.03	*p* = 0.05
V̇_I_ (L/min)	11.0 ± 3.0	14.3 ± 3.2	*p* < 0.0001
SSCD (a.u.)	32.3 ± 9.3	46.7 ± 13.0	*p* < 0.0001
Urine pH	5.99 ± 0.5	5.82 ± 0.2	*p* = 0.2
AMS Score	0 (0–1)	0 (0–2)	*n* not sufficient
**Group**	**Acetazolamide Group (*n* = 11)**		
HR (min^−1^)	77.0 ± 8.3	78.2 ± 15.0	*p* = 0.8
MAP (mmHg)	87.6 ± 9.3	95.0 ± 8.7*	*p* = 0.01
[Hb] (g/L)	138.3 ± 10.8	150.3 ± 10.6*	*p* = 0.02
CaO_2_ (ml/dl)	18.2 ± 1.4	17.9 ± 1.2	*p* = 0.56
P_ET_CO_2_ (mmHg)	30.7 ± 2.2	24.7 ± 1.5*	*p* < 0.0001
SpO_2_ (%)	96.9 ± 1.1	87.7 ± 2.8*	*p* < 0.0001
SI (a.u.)	0.32 ± 0.02	0.28 ± 0.02*	*p* = 0.0005
V̇_I_ (L/min)	11.3 ± 1.4	16.9 ± 2.9*	*p* = 0.0001
SSCD (a.u.)	35.8 ± 4.8	60.7 ± 12.9*	*p* = 0.0001
Urine pH	6.06 ± 0.7	6.68 ± 0.5*	*p* = 0.04
AMS Score	0 (0–1)	1 (0–1)	*n* not sufficient

#### 2.2.2 Acetazolamide use

Following an assessment of VA with ascent in the complete group (*n* = 25), we subsequently compared two subgroups based on known acetazolamide status. All expedition participants independently obtained a personal supply of acetazolamide via a prescription from their physician prior to the departure for the expeditions, as per accepted guidelines (e.g., Luks et al., 2019). For the expeditions where participants were not taking oral prophylactic acetazolamide (NAz), it was available for treatment of AMS symptoms under the guidance of the organization team, as needed. The NAz group did not take prophylactic acetazolamide nor corticosteroids for treatment of AMS at any time during the incremental ascent to 4240 m. For the expedition where participants were taking acetazolamide (Az), participants were self‐administering an oral prophylactic dose (125 mg BID) during ascent. The Az group began taking acetazolamide on day one of ascent from 1400 to 2840 m (flight to Lukla airport and first trekking day), and continued self‐administration twice daily (morning and night) as per instructions from the expedition organizers during trekking at altitude (e.g., Basnyat et al., 2003; Luks et al., 2019; van Patot et al., 2008).

This observational retrospective evaluation between NAz and Az groups was not planned a priori. Rather, the unique opportunity to assess the potential effects of a self‐administered oral prophylactic dose of acetazolamide on VA arose post hoc, given that data collection occurred across a number of HA research expeditions, where one group was taking oral acetazolamide as a part of the safety precautions of the expedition, and another group was not. Given that (a) this was not a drug intervention study (i.e., clinical trial) and the acetazolamide was obtained by each participant individually in advance via prescription from their own personal physicians and (b) the drug use took place outside of Canada (Nepal), Health Canada approval was not required, as it is outside the scope of Part C, Division 5 of the Food and Drug Regulations, and thus a Clinical Trial Application was not required to be submitted for review (Health Canada, *personal communication*).

#### 2.2.3 Daily ancillary measurements

Resting ancillary physiological measurements were taken every morning between 06:00 and 08:00 local time following one night at each altitude: 1130/1400 m (day zero) and 4240 m (day seven) to characterize steady‐state physiological responses to incremental ascent to altitude (Table 1). All ancillary physiological measures were obtained at rest in a seated position following >2‐min rest with eyes closed and white noise played through headphones to limit distraction. Brachial arterial blood pressure was measured via an automated blood pressure monitor (model BP786n; Omron). Mean arterial pressure (MAP) was calculated as a weighted mean (1/3 systolic +2/3 diastolic). Hemoglobin concentration [Hb] was obtained via finger capillary blood sample using sterile lancets (AccuChek, Softclix) with standard practice and universal precautions, measured via hemoglobinometer (Hemocue Hemoglobin System, Hb201+ with microcuvettes). Oxygen content (CaO_2_) was subsequently calculated as 1.36 × [Hb] × SpO_2_/100, where 1.36 is the binding capacity of oxygen to hemoglobin (Hüfner's constant), [Hb] is hemoglobin concentration (g/dl) and SpO_2_ is peripheral arterial oxygen saturation (%; see below). Given we did not have PaO_2_, CaO_2_ was estimated without it (using only [Hb] and SpO_2_), given the negligible contribution of PaO_2_ when [Hb] is normal or high.

For consistency in comparison, all baseline P_ET_CO_2_ values were obtained at 1400 m for SSCD calculations. Mainstream P_ET_CO_2_ (mmHg; atmospheric pressure adjusted) was measured using a portable capnograph (EMMA, Masimo) and a personal mouthpiece and nose clip, obtained from a running average after steady‐state was achieved. The portable capnograph utilized for P_ET_CO_2_ measures is rated for accuracy to an atmospheric pressure equivalent to approximately P_ATM_ of ~525 mmHg (~3200 m; https://bit.ly/3zqWQeJ). In a previous study, we noted that (a) P_ET_CO_2_ using this model of capnograph underestimated PaCO_2_ with ascent and (b) the underestimation of PaCO_2_ by P_ET_CO_2_ was exaggerated with ascent to 5160 m following an identical ascent profile (i.e., values diverged with ascent; see Zouboules et al., 2018); Correction model is published in (Isakovich et al., n.d.). Specifically, the following values were added to P_ET_CO_2_ values obtained from the portable capnograph, within‐individual: 1400 m, +0.405 mmHg; 4240 m, +4.665 mmHg (see Isakovich et al., n.d.).

Morning urine pH measurements were obtained on all participants and are utilized here post hoc to confirm acetazolamide status on measurement days (i.e., day zero at 1130/1400 m, and day seven at 4240 m). Participants provided a sample of their first‐morning urination into a new, clean 110 ml sample container with a screw cap that could be secured immediately following collection and analyzed within 5–30 min. Urine pH was measured aerobically using a pH meter and biological probe (B10P; VWR; sympHony), calibrated daily using standard pH buffers (3 and 7), and automatically temperature corrected.

**TABLE 2:** Resting ancillary and cardiorespiratory variables following incremental ascent to altitude at 4240 m. Values are reported for the combined participant group (*n* = 34) and each subgroup, both the no acetazolamide (NAz; *n* = 22) and acetazolamide groups (Az; *n* = 12). P_ATM_, atmospheric pressure (calculated; see Section 2); P_I_O_2_, pressure of inspired oxygen ([P_ATM_‐47] × 0.21); HR, heart rate; MAP, mean arterial pressure; [Hb], hemoglobin concentration; CaO_2_, oxygen content; P_ET_CO_2_, pressure of end‐tidal CO_2_ (corrected, see Section 2); SpO_2_, peripheral arterial oxygen saturation; V̇_I_, minute ventilation; SSCD, steady‐state chemoreflex drive (V̇_I_/SI); see Section 2). MAP, P_ET_CO_2_, urine pH, and AMS scores from morning ancillary measures (06:00–08:00), after one night‘s sleep after arrival at 4240 m. All other measures from LabChart data during rest day at each altitude (09:00–17:00). Statistical tests utilized were two‐tailed paired *t*‐tests comparing NAz and Az groups at 4240 m for each variable, with *p*‐values listed for each test. For AMS scores, a non‐parametric Mann–Whitney *U* test was utilized. Asterisk (*) indicates a significant difference from NAz (*p* < 0.05). Values are reported as mean ± standard deviation, except for AMS scores, which are reported as median (range)

Variable/location	4.240 m (Day 7)			*p*‐value (NAz vs. Az)
P_ATM_ (mmHg)	460			N/A
P_I_O_2_ (mmHg)	87			N/A

**Group**	**All (*n* = 34)**	**NAz (*n* = 22)**	**Az (*n* = 12)**	
HR (min^−1^)	75.9 ± 14.6	75.3 ± 14.7	76.8 ± 15.1	*p* = 0.78
MAP (mmHg)	98.0 ± 8.9	100.2 ± 8.1	93.8 ± 9.2*	*p* = 0.04
[Hb] (g/L)	148.4 ± 11.8	147.6 ± 12.7	149.8 ± 10.2	*p* = 0.62
CaO_2_ (ml/dl)	17.4 ± 1.5	17.2 ± 1.7	17.8 ± 1.2	*p* = 0.31
P_ET_CO_2_ (mmHg)	26.1 ± 2.5	26.9 v2.6	24.7 ± 1.4*	*p* = 0.009
SpO_2_ (%)	86.4 ± 2.7	85.9 ± 2.7	87.4 ± 2.8	*p* = 0.12
SI (a.u.)	0.30 ± 0.03	0.31 ± 0.03	0.28 ± 0.02*	*p* = 0.006
V̇_I_ (L/min)	14.7 ± 3.3	13.6 ± 2.9	16.7 ± 2.9*	*p* = 0.005
SSCD (a.u.)	49.5 ± 14.1	43.9 ± 11.7	59.6 ± 12.8*	*p* = 0.001
Urine pH	6.27 ± 0.57	6.03 ± 0.48	6.70 ± 0.45*	*p* = 0.0004
AMS Score	0 (0–2)	0 (0–2)	1 (0–2)	*p* = 0.43

Lastly, self‐reported AMS scores were obtained using the updated Lake Louise Questionnaire (Roach et al., 2018). Unlike ventilation and SpO_2_ data (described below), individual P_ET_CO_2_, HR, MAP, [Hb], and AMS scores were documented by hand.

#### 2.2.4 Measurement of resting ventilation

Minute ventilation was measured with participants in a seated position in a dark, quiet laboratory (Calgary, 1130 m) or lodge bedroom (Kathmandu at 1400 m or Pheriche at 4240 m) between the hours of 10:00 and 17:00 on rest days following one night sleep at the respective altitude. Participants kept their eyes closed throughout the testing protocol and wore ear plugs to minimize distraction. Participants were instrumented with a personal mouthpiece, nose clip and bacteriological filter, proximal to the flow head. Following instrumentation, we collected 10‐min of baseline data for each participant, using a 16‐channel PowerLab system (Powerlab/16SP ML880; AD Instruments; ADI) and analyzed offline using commercially available software (LabChart Pro software 8.0). Respiratory flow was measured via pneumotachometer (800 L flow head and spirometer amplifier; Hans Rudolph and ADI ML141; calibrated with a 3 L syringe daily). The dead space associated with the mouthpiece, bacteriological filter, and flow head was the same between experimental conditions between expeditions. Instantaneous minute ventilation (V̇_I_, L/min) was calculated as the product of breath‐by‐breath inspired volume (V_TI_; calculated from the integral of the flow signal) and respiratory rate (R_R_, min^−1^; calculated by 60/period of the flow signal). Continuous measures of SpO_2_ were obtained by pulse oximeter (ADI ML320) placed on the right middle finger.

ADI LabChart data were digitally archived for later offline analysis, and measures were derived from a representative 2‐minute steady‐state average (sampled each second; ~120 samples) near the end of each 10‐min baseline period, analyzed by the same team member for consistency (T.L.M). Given the known effects of mouthpiece and nose clip use on eliciting relative hyperventilation in some participants at rest (e.g., Askanazi et al., 1980; Sackner et al., 1980; Scott, 1993; Weissman et al., 1984), we only included participants in the final analysis that increased minute ventilation between 1130/1400 m (day 0) and 4240 m (day 7) for delta responses with ascent, representing the expected ventilatory acclimatization with ascent over 7 days, given that all participants were hypocapnic with ascent, as expected.

For transparency, although we initially recruited 36 participants at low altitude, two declined to have measures obtained at 4240 m, and nine participants were excluded in the before‐after ascent comparison due to relative hyperventilation during baseline at 1130/1400 m (see Section 4), for a final participant pool of *n* = 25, with subgroups of *n* = 14 in the NAz subgroup and *n* = 11 in the Az subgroup. These participants were pooled to assess the large group change in SSCD (see below), as well as compared between them (i.e., NAz vs. Az) with ascent. However, we included all available participants for an absolute comparison at 4240 m between NAz (*n* = 22) and Az (*n* = 12) groups to confirm our findings.

Note that baseline V̇_I_ and SpO_2_ for the Az group (*n* = 11) and half of the NAz group (*n* = 7), values were obtained at 1130 m (P_I_O_2_ ~ 130 mmHg), with these data from the remaining participants in the NAz group (*n* = 7) obtained at 1400 m (P_I_O_2_ ~ 125 mmHg). There were no statistical differences in V̇_I_ (*p* = 0.24) and SpO_2_ (*p* = 0.42) between participants at the two baseline locations (1130 m vs. 1400 m), and thus data were pooled to represent pre‐ascent baseline in the larger group.

#### 2.2.5 Steady‐state chemoreflex drive during ascent

Minute ventilation, P_ET_CO_2_, and SpO_2_ measurements were performed on rest days in Calgary (1130 m) or Kathmandu (1400 m; day zero), and again in Pheriche (4240 m) to assess the magnitude of SSCD following incremental ascent. In order to calculate the SSCD, a stimulus index (SI; P_ET_CO_2_/SpO_2_) was first calculated, whereby ventilation is known to be linearly and directly proportional to changes in P_ET_CO_2_ (e.g., Duffin, 2011; Nielsen & Smith, 1952) and linearly and inversely proportional to changes in SpO_2_ (e.g., Rebuck & Campbell, 1974). Ventilation was then indexed to the SI to calculate SSCD (see Bruce et al., 2018; Leacy et al., 2021; Pfoh et al., 2017) to assess the integrative contributions of chemostimuli to ventilation in the steady‐state.

(2) StimulusIndexSI=PETCO2/SpO2a.u.SSCD=V˙I/SIa.u..

#### 2.2.6 Statistical analysis

Table 1 presents cardiorespiratory and ancillary data during ascent as mean ± standard deviation (SD) in the combined group of participants (*n* = 25), and for separate subgroups (NAz [*n* = 14] vs. Az [*n* = 11]). Self‐reported AMS scores within groups with ascent were assessed using non‐parametric Wilcoxon signed‐rank tests (i.e., paired; Table 1).

**FIGURE 1:**
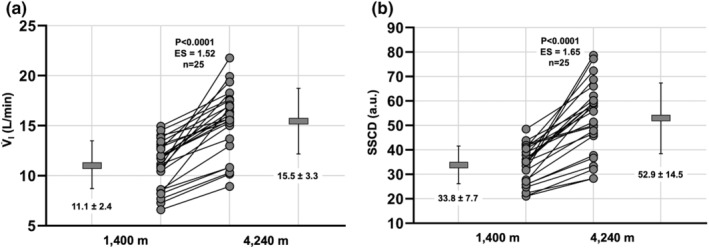
Ventilatory acclimatization with ascent to 4240 m over 7 days in the combined participant group (*n* = 25). (a) Minute ventilation (V̇_I_) prior to (1130/1400 m and following ascent to 4240 m. (b) Steady‐state chemoreflex drive (SSCD) prior to and following ascent to 4240 m. Individual participants (gray circles) are connected for within‐individual comparisons. Gray dash and error bars represent mean (values reported on graph) and standard deviation. Individual *p*‐values are reported on each graph, where *p* < 0.05 denotes statistical difference from baseline (1130/1400 m). ES, effect size calculated via Hedges' *g*.

**FIGURE 2:**
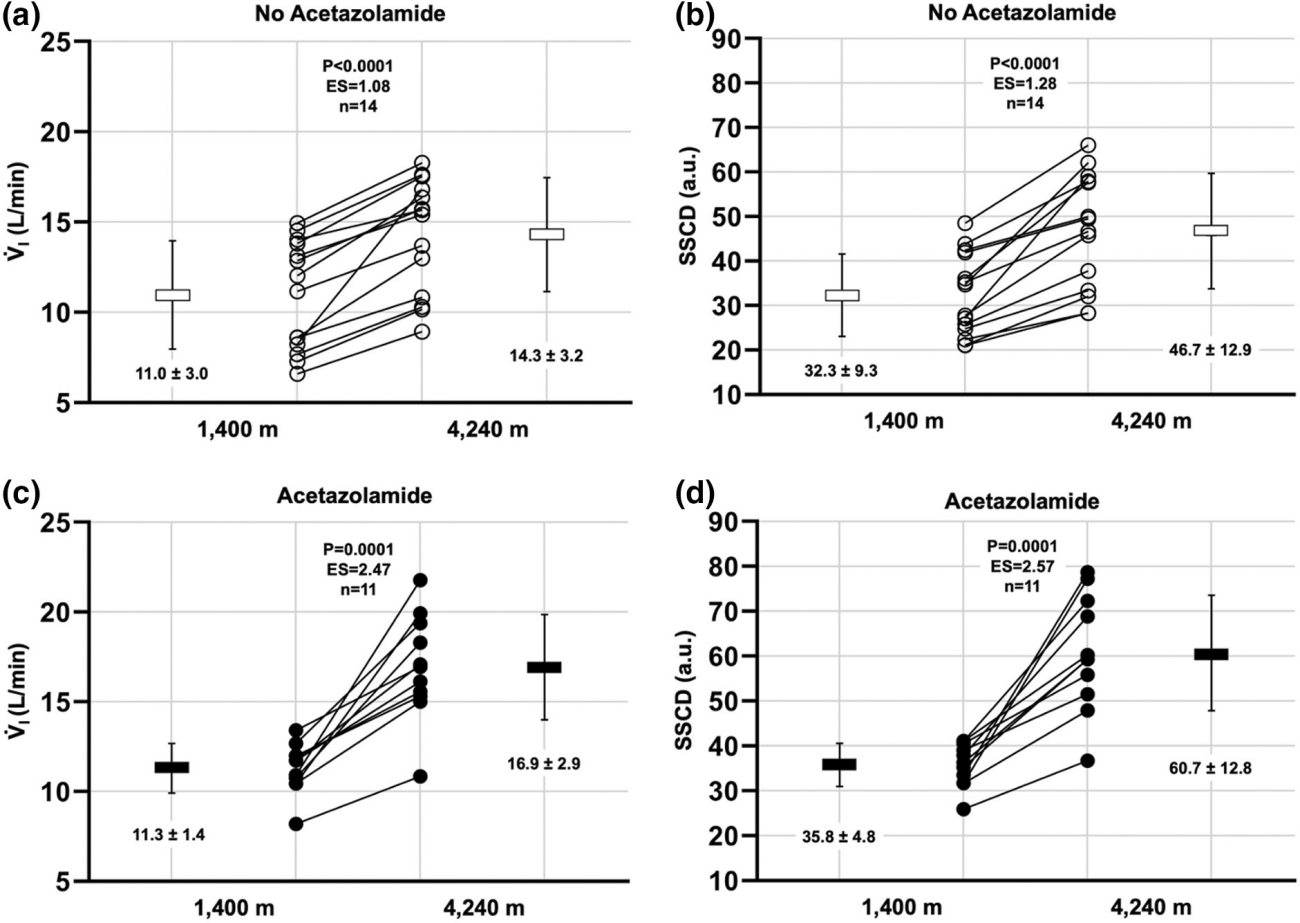
Ventilatory acclimatization before and after ascent to 4240 m over 7 days without and with prophylactic oral acetazolamide. (a) Minute ventilation (V̇_I_) prior to (1130/1400 m) and following ascent to 4240 m in the no acetazolamide (NAz) group (*n* = 14). (b) Steady‐state chemoreflex drive (SSCD) prior to and following ascent to 4240 m in the NAz group (*n* = 14). (c) Minute ventilation (V̇_I_) prior to and following ascent to 4240 m in the acetazolamide (Az) group (*n* = 11). (d) SSCD prior to and following ascent to 4240 m in the Az group (*n* = 11). White circles, individual participants in the NAz group (panels a and b). Black circles, individual participants in the Az group (panels c and d). Individual participants are connected for within‐individual comparisons.

To compare variables between 1130/1400 m (day zero) and 4240 m (day seven) in the combined group (*n* = 25), as well as within separate NAz (*n* = 14) and Az (*n* = 11) groups with ascent, we performed two‐tailed, paired t‐tests (Table 1; Figures 1a,b and 2a,b).

**FIGURE 3:**
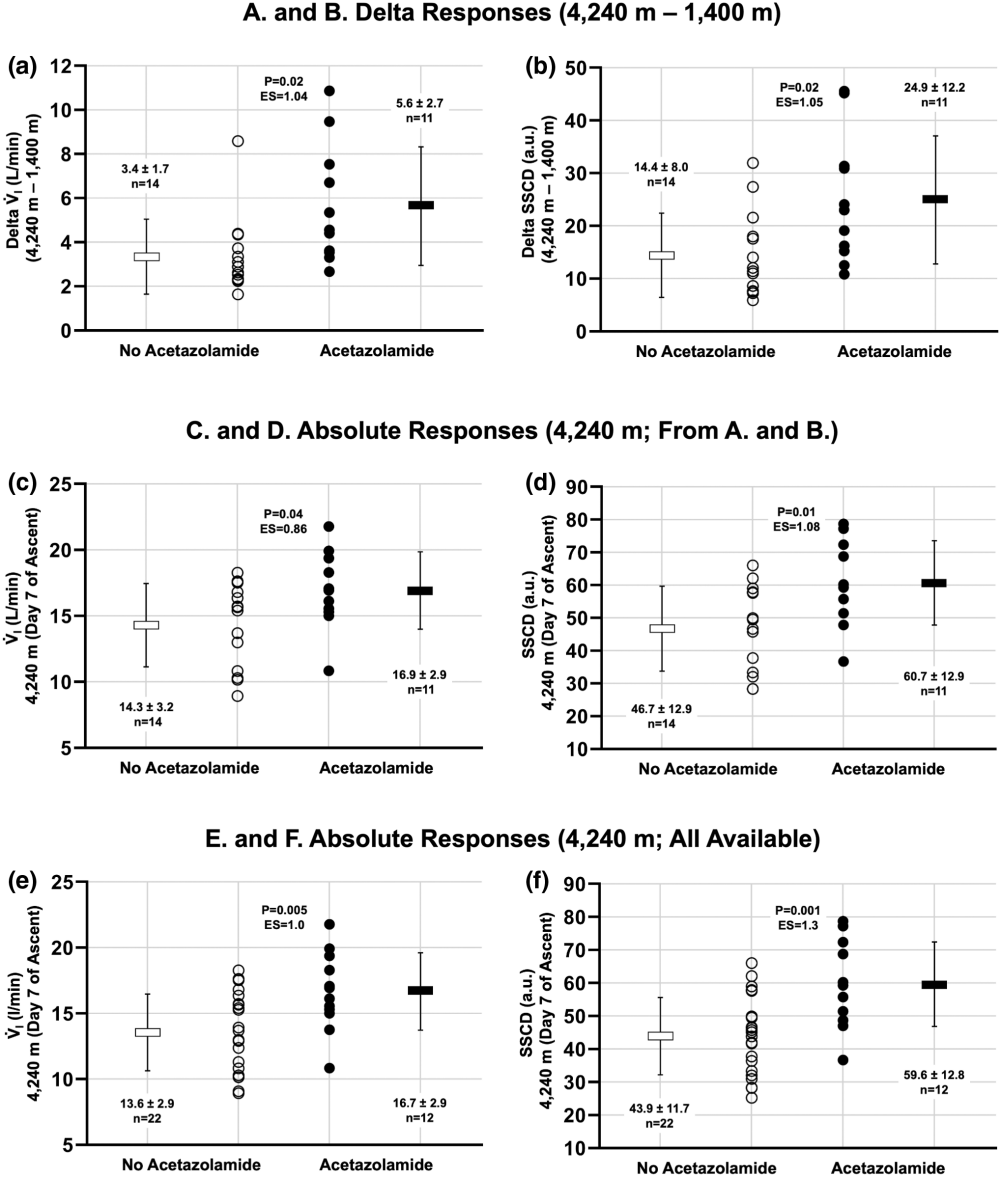
Comparison of participants with and without oral acetazolamide in metrics of ventilatory acclimatization following ascent to 4240 m over 7 days. (a) Comparison of delta V̇_I_ (4240–1400 m) between no acetazolamide (NAz; *n* = 14) and acetazolamide (Az; *n* = 11) subgroups with ascent. (b) Comparison of delta SSCD between NAz and Az subgroups with ascent. (c) Comparison of absolute minute ventilation (V̇_I_) following ascent to 4240 m in the NAz; versus Az subgroups. (d) Comparison of absolute steady‐state chemoreflex drive (SSCD) following ascent to 4240 m in the versus Az subgroups. These participants in a–c are from those that were compared with 1130/1400 m (i.e., before and after ascent; *n* = 25; see Figures 1 and 2). (e) Comparison of absolute minute ventilation (V̇_I_) following ascent to 4240 m in the no acetazolamide (NAz; *n* = 22) versus acetazolamide (Az; *n* = 12) groups. (d) Comparison of absolute steady‐state chemoreflex drive (SSCD) following ascent to 4240 m in the no acetazolamide (NAz; *n* = 22) versus acetazolamide (Az; *n* = 12) groups. In (e and f), these values are from every available participant measurement made at 4240 m (*n* = 34). White circles, individual participants in the NAz group. Black circles, individual participants in the Az group. Dash and error bars represent mean and standard deviation (values reported on graph). Individual *p*‐values and effect sizes (ES) are reported on each graph, where *p* < 0.05 denotes a statistical difference between NAz and Az groups. ES, effect size calculated via Hedges' *g*.

In addition, to compare the within‐individual change (delta) in V̇_I_ and SSCD between NAz and Az groups, we performed two‐tailed, paired t‐tests on the absolute within‐individual change (Figure 3a,b).

For direct comparison between NAz (*n* = 14) and Az (*n* = 11) groups at 4240 m, we compared the absolute difference in V̇_I_ and SSCD between NAz and Az groups using two‐tailed, unpaired t‐tests (Figure 3c,d). In addition, as a further comparison of absolute values at 4240 m between groups (i.e., not only those compared before and after ascent in Figures 1, 2, 3), a larger group of available participants (*n* = 34) was compared between NAz (*n* = 22) and Az (*n* = 12) at 4240 m using two‐tailed, unpaired *t*‐tests (Figure 3e,f).

Lastly, the relationship between self‐reported AMS scores with ascent to 4240 m was assessed using a Spearman Rho (*r*_s_) correlation. In addition, potential differences in AMS scores between NAz and Az subgroups at 4240 m were assessed using non‐parametric Mann–Whitney *U* tests (i.e., unpaired).

The effect size of ventilatory and SSCD responses with ascent was assessed via Hedges' *g* tests (Figures 1, 2, 3). Statistical tests were performed using GraphPad Prism (v9.1.2). In all cases, statistical significance was assumed at *p* < 0.05.

## 3 RESULTS

### 3.1 Participant demographics

All participants included in the final analysis followed an identical ascent profile (see Section 2), specifically ascending from 1400 to 4240 m over 6 days, with measures made in day seven at 4240 m.

To characterize responses during ascent, only participants that demonstrated an increase in ventilation with identical ascent profiles from 1400 to 4240 m were included in the analysis (*n* = 25; 15 females; age 24.9 ± 6.9 years, BMI 23.8 ± 3.1 kg/m^2^). A total of 14 (7 females; age 24.1 ± 6.6 years; BMI 23.8 ± 2.5 kg/m^2^) and 11 participants (8 females; age 25.9 ± 7.5 years; BMI of 23.9 ± 3.8 kg/m^2^) were included in the NAz and Az groups, respectively.

To confirm the effect of oral Az between subgroups only at 4240 m (i.e., not comparing deltas from baseline at low altitude), a total of 34 participants were included in the analysis (23 females; age 24.1 ± 6.1 years, BMI 23.7 ± 3.1 kg/m^2^). Specifically, a total of 22 participants (14 females; age 23.1 ± 5.4 years; BMI 23.6 ± 2.8 kg/m^2^) and 12 participants (9 females; age 26.0 ± 7.1 years; BMI of 23.8 ± 3.7 kg/m^2^) were included in the NAz and Az groups, respectively.

### 3.2 Ancillary variables

Cardiorespiratory and ancillary variables with ascent from 1130/1400 m to 4240 m over 7 days in the total group (*n* = 25), and subsequently for each of the NAz (*n* = 14) and Az (*n* = 11) subgroups are reported in Table 1. Note that baseline data prior to ascent in both the NAz and Az groups were acetazolamide‐free. As expected, P_ET_CO_2_ and SpO_2_ decreased with ascent, whereas V̇_I_ and SSCD increased with ascent in both groups. Aerobic urine pH was unchanged with ascent in the NAz group, but was significantly more alkaline in the Az group, confirming that the participants were self‐administering acetazolamide, which elicited an expected renal effect. Lastly, although self‐reported AMS scores increased with ascent in both NAz and Az groups, there was no statistical difference between NAz and Az groups in at 4240 m on day seven following 6 days of ascent.

### 3.3 Ventilation and steady‐state chemoreflex drive (SSCD) during ascent to altitude

In the combined participant group with ascent, V̇_I_ increased significantly with ascent (~39%, *p* < 0.0001, Hedges' *g* = 1.52; Figure 1a). Similarly, SSCD increased significantly with ascent (~56%, *p* < 0.0001, Hedges' *g* = 1.65; Figure 1b).

When assessing the NAz and Az groups separately, V̇_I_ increased significantly with ascent in the NAz group (~31%, *p* < 0.0001, Hedges' *g* = 1.08; Figure 2a) and Az group (~50%, *p* < 0.0001, Hedges' *g* = 2.47; Figure 2c). Similarly, SSCD increased significantly with ascent in the NAz group (~45%, *p* < 0.0001, Hedges' *g* = 1.28; Figure 2b) and the Az group (~70%, *p* < 0.0001, Hedges' *g* = 2.57, respectively; Figure 2d).

When comparing delta V̇_I_ (4240–1400 m) between NAz and Az subgroups with ascent, the Az group increased more than the NAz group (*p* = 0.02, Hedges' *g* = 1.04; Figure 3a). Similarly, when comparing delta SSCD between NAz and Az subgroups with ascent, the Az group increased more than the NAz group (*p* = 0.02, Hedges' *g* = 1.05; Figure 3b).

When comparing absolute values at 4240 m in participants included in Figure 2 (*n* = 25) between NAz (*n* = 14) and Az (*n* = 11) subgroups, V̇_I_ was larger in the Az group versus NAz group (*p* = 0.04, Hedges' *g* = 0.86; Figure 3c). Similarly, SSCD was larger in the Az group versus NAz group (*p* = 0.01, Hedges' *g* = 1.08; Figure 3d).

Lastly, when comparing absolute values at 4240 m in all recruited participants (*n* = 34; i.e., not only those reported in Figures 1, 2, 3) between NAz (*n* = 22) and Az (*n* = 12) subgroups, V̇_I_ was larger in the Az group versus NAz group (*p* = 0.005, Hedges' *g* = 1.0). Similarly, SSCD was larger in the Az group versus NAz group (*p* = 0.001, Hedges' *g* = 1.3). This additional comparison in a larger group of available participants confirms the responses noted in Figure 3a–d.

### 3.4 Relationship with AMS scores

Self‐reported AMS scores were significantly higher at 4240 m (*p* < 0.05) compared to 1400 m (*n* = 25). However, there was no relationship between SSCD and self‐reported AMS scores at 4240 m in the large group (*n* = 34; *r*_s_ = 0.11, *p* = 0.52). In addition, there were no differences between self‐reported AMS scores at 4240 m between NAz (*n* = 22) and Az (*n* = 12) groups at 4240 m (*p* = 0.43).

## 4 DISCUSSION

We aimed to characterize VA through a novel index of SSCD during incremental ascent to HA in a large cohort of lowlanders ascending to 4240 m over 7 days, and subsequently assess the superimposed effects of oral acetazolamide by comparing two separate subgroups, one not taking acetazolamide (NAz) and one taking an oral prophylactic dose of acetazolamide (Az; 125 mg BID) as a part of the expedition organization. The principal findings of our methodological study include: (a) ascent to HA over 7 days elicited an increase in V̇_I_ and SSCD following ascent to 4240 m, suggesting that the SSCD metric captures VA with ascent, and (b) the Az subgroup had significantly higher V̇_I_ and SSCD than the NAz, both in delta responses from low altitude and absolute values at 4240 m. However, there was no relationship between SSCD and self‐reported AMS scores following ascent to 4240 m, nor were there differences between NAz and Az subgroups in AMS symptom severity, likely due to the low reported scores with incremental ascent. These results suggest that although VA was apparent following a 7‐day time‐course with incremental ascent, an oral 125 mg BID prophylactic oral dose of acetazolamide can play an important role in augmenting VA during incremental ascent to 4240 m.

### 4.1 Testing respiratory chemoreflexes at altitude

The chemoreflex control of breathing is mediated through two separate but converging feedback loops (Duffin & Mahamed, 2003). The central respiratory chemoreceptors respond to an accumulation or reduction of metabolically derived CO_2_ and/or [H^+^] within chemosensitive brainstem neurons and glia, eliciting a central chemoreflex (Duffin, 2010; Guyenet et al., 2010; Nattie & Li, 2012; Pfoh et al., 2016). The peripheral respiratory chemoreceptors are stimulated by low PO_2_ (hypoxemia), eliciting a corresponding increase in ventilation (i.e., HVR; Duffin, 2011; Teppema & Dahan, 2010), which contributes to the maintenance of blood‐gas homeostasis, improving oxygenation in the context of acute or chronic hypoxia. Interestingly, the acute HVR is polyphasic, where a reduction in ventilation (hypoxic ventilatory decline; HVD) shortly follows the transient peak response in ventilation (Powell et al., 1998; Sato et al., 1994; Steinback & Poulin, 2007), and hyperventilation‐induced hypocapnia persists (i.e., poikilocapnic hypoxia), further blunting the HVR (e.g., Steinback & Poulin, 2007), and reducing central chemoreceptor stimulation. If the hypoxic challenge is sustained (i.e., HA), the HVR is increased in magnitude through carotid body plasticity (e.g., Robbins, 2007; Sato et al., 1992), and the sustained hypocapnia ultimately leads to renally mediated metabolic compensatory mechanisms (Ainslie et al., 2013; Krapf et al., 1991; Zouboules et al., 2018), with the metabolic acidosis likely stimulating both central and peripheral chemoreceptors at rest. Thus, as both central and peripheral chemoreflex magnitude are increased with sustained exposure to hypoxia, assessing respiratory chemoreflexes and VA is of interest in HA fieldwork settings (Bruce et al., 2018; Dempsey et al., 2014; Leacy et al., 2021; Sato et al., 1992; Smith et al., 2017). Usually, the peripheral chemoreflex is the target of interest via testing the transient HVR test (e.g., Teppema & Dahan, 2010), despite the simultaneous alterations in and contributions from the central chemoreflex to ventilation with sustained exposure to HA.

### 4.2 Laboratory HVR test utility and caveats

Many methodological perspectives exist on how best to assess the HVR (e.g., Duffin, 2011; Pfoh et al., 2016, 2017; Powell et al., 1998; Teppema & Dahan, 2010) including rebreathing, steady‐state and transient hypoxic tests to elicit a peripheral chemoreflex. Rebreathing or steady‐state hypoxic tests involve exposing a participant to various fractions of inspired oxygen using chambers, small rebreathing bags or large Douglas bags pre‐filled with mixed gasses, or dynamic end‐tidal forcing systems, over several minutes (Teppema & Dahan, 2010). Alternatively, transient hypoxic tests expose participants to short bouts of hypoxia (i.e., one or more breaths) via administration of 100% N_2_ (e.g., Milloy et al., 2022; Pfoh et al., 2016; Teppema & Dahan, 2010). In both cases, the HVR is often quantified by the initial peak in ventilation following the acute hypoxic stimulus. Although the HVR can readily be tested in laboratory contexts, these transient HVR tests lack feasibility in HA fieldwork contexts for a number of reasons including: (a) limited equipment portability, (b) the risk associated with acute hypoxia in participants who are already chronically hypoxic, (c) the effects of a hypoxic ventilatory decline and hypocapnia that occur with both acute (i.e., steady‐state tests) and chronic (i.e., HA) hypoxic exposure, and (d) the inability to isolate peripheral and/or central chemoreflexes during the protocol due to a lack of stimulus specificity and/or interactions between chemoreceptor compartments (e.g., Wilson & Teppema, 2016). Lastly, HVR tested via differential methods have been shown to be poorly or not correlated in magnitude within individuals (Pfoh et al., 2016, 2017). For example, we recently tested the effects of a number of transient and steady‐state HVR tests in unacclimatized participants in a laboratory setting, and found that the within‐individual magnitude was not correlated between tests, nor was the magnitude related to oxygenation while breathing steady‐state hypoxia (F_I_O_2_ 0.13–0.14), suggesting that specific tests of the HVR are not capturing the ventilatory strategy utilized to protect blood gases in steady‐state hypoxia (Pfoh et al., 2016, 2017). These matters of feasibility, consistency, and utility have driven the development of a portable method and associated metric that can be applied consistently in laboratory and fieldwork contexts, to capture the integrated ventilatory response to prevailing chemostimuli in the steady‐state.

### 4.3 Steady‐state chemoreflex drive (SSCD) with ascent: Effect of acetazolamide

In an attempt to address the many caveats associated with HVR tests, particularly with ascent to HA, we previously developed and characterized a novel index of steady‐state chemoreflex drive, which assesses the relationship of steady‐state ventilation at prevailing chemostimuli (i.e., CO_2_ and O_2_), acting on both central and peripheral chemoreceptors. We found that the SSCD did not change from breathing ambient air to breathing 20‐min of steady‐state hypoxia (F_I_O_2_ 0.13–0.14; Pfoh et al., 2017), likely due to the antagonistic effects of hypoxia and concomitant hypocapnia (e.g., poikilocapnic hypoxia; e.g., Steinback & Poulin, 2007). However, we previously showed that (a) SSCD increased in a small group of lowlanders following 10 days of incremental ascent to 5160 m (Bruce et al., 2018), (b) increases with rapid ascent and residence at 3800 m (Bird, Leacy, et al., 2021) and (c) SSCD hysteresis with incremental ascent to and descent from 5160 m quantifies VA, the magnitude of which has inverse effects on AMS severity (Leacy et al., 2021). Here, we advance on these characterizations by assessing the effects of incremental ascent on ventilation and SSCD in a large group of participants over 7 days, and subsequently comparing separate groups with and without an oral prophylactic dose of acetazolamide. We propose that the SSCD method overcomes many of the caveats associated with peak response HVR tests and captures the integrated central and peripheral chemoreflex drive in the steady‐state, with utility in assessing VA during fieldwork at HA. In the present study, we established that SSCD is augmented with 7 days of incremental ascent to 4240 m (i.e., it captures VA), which is further augmented when superimposed with oral acetazolamide.

### 4.4 Acute mountain sickness with ascent: Effect of acetazolamide

Many experimental studies and reviews have assessed the efficacy of a prophylactic oral dose of acetazolamide in preventing and/or reducing AMS symptoms (Basnyat et al., 2003; Gao et al., 2021; Kayser et al., 2012; Lipman et al., 2020; Low et al., 2012; McIntosh et al., 2019; Nieto Estrada et al., 2017; van Patot et al., 2008) with mixed results. There are several limitations with drawing conclusions from these studies, in part due to the subjective nature of AMS reporting, the site of recruitment (e.g., low vs. high altitude), and different ascent profiles (e.g., rapid vs. incremental ascent). The prophylactic oral Az dose did not reduce self‐reported AMS symptoms following 7 days of incremental ascent, likely due to the incremental nature of our ascent model, and the relatively low reported scores. This is in contrast to a previous study (Burtscher et al., 2014), where a prophylactic dose of acetazolamide prior to ascent improved AMS scores immediately following rapid ascent to 3480 m compared to placebo, suggesting that ascent profile (i.e., rapid vs. incremental), duration of stay and absolute altitude may affect the potential utility of prophylactic acetazolamide. Additionally, the recommended lowest prophylactic dose is varied (e.g., Luks et al., 2019 vs. Dumont et al., 2000), with the potential to be reduced further (e.g., McIntosh et al., 2019). Acetazolamide elicits dose‐dependent side effects, including polyuria, paraesthesia, fatigue, and dysgeusia (Schmickl et al., 2020), and thus there is a desire to find the lowest prophylactic dose that minimizes both drug side effects and reported AMS symptoms. Although it has been well‐established that a prophylactic dose is superior to placebo in limiting AMS symptoms (Gao et al., 2021; Kayser et al., 2012; Low et al., 2012), the findings in the present study suggest no difference in AMS scores between NAz and Az groups using identical incremental ascent profiles, which was contrary to our initial hypothesis. The similarity in AMS scores between our NAz and Az groups with ascent may be attributed in part to the fact that (a) the two groups were different groups across different expeditions (i.e., not randomized or within‐individual comparisons) and/or (b) both groups had a low risk for developing AMS given the slow, incremental ascent profile we utilized (e.g., Imray, 2012; Kayser et al., 2012; Luks et al., 2019).

Participants in the Az group were instructed to self‐administer a prophylactic dose of acetazolamide starting on day one of ascent (125 mg BID; 250 mg/day) as a part of the safety precautions of the expedition organization. We subsequently confirmed that these participants were taking acetazolamide by assessing urine pH measures, in comparison with the NAz group. The urine pH in the NAz group was unaffected with ascent, despite incremental hypocapnia, likely due to the aerobic nature of these urine measurements compared to other studies that used anaerobic samples (e.g., Galdston, 1955; Ge et al., 2006; Gledhill et al., 1975). However, urine pH became more alkaline in the Az group, confirming increased HCO_3_^−^ excretion and/or H^+^ retention (i.e., metabolic acidosis) in response to oral acetazolamide (e.g., Galdston, 1955; Leaf & Goldfarb, 2007). We conclude that both V̇_I_ and SSCD responses are augmented with incremental ascent to 4240 m, suggesting beneficial effects of low‐dose oral acetazolamide, even during low‐risk incremental ascent models, at least to ~4000 m. We anticipate that larger doses (e.g., AMS treatment doses; 500 mg/day or larger; e.g., Basnyat & Murdoch, 2003; Luks et al., 2019) would further stimulate V̇_I_ and SSCD with ascent, but this remains to be systematically tested.

### 4.5 Methodological considerations

Our study is in agreement with previous work from our group assessing SSCD with ascent (Berthelsen et al., 2022; Bird, Kalker, et al., 2021; Bird, Leacy, et al., 2021; Bruce et al., 2018; Leacy et al., 2021). In the present study, we clearly demonstrate that SSCD captures VA with incremental ascent to 4240 m over 7 days in a large group of lowlanders. In addition, we found that the use of prophylactic oral acetazolamide augmented VA, quantified via the SSCD index. This is the most comprehensive assessment of the SSCD metric in participants ascending to 4240 m, as a method to characterize VA, with a prophylactic oral dose of Az augmenting this response.

Although the SSCD metric holds promise as a potential tool to integrate into field studies assessing respiratory control with ascent to altitude, our methodological study has a number of limitations worthy of consideration. First, although we initially recruited 36 participants for this methodological study, a reduced number were included in the final analysis. Specifically, two opted out of measures at 4240 m due to symptoms of altitude‐related illness. Additionally, nine were excluded post hoc due to relative hyperventilation at low altitude, whereby their ventilation was higher at 1130/1400 m (day zero) compared to 4240 m, which is physiologically incongruent with exposure to sustained hypobaric hypoxia and the hyperventilation‐induced hypocapnia that was apparent in all participants with ascent. Although we utilized a calibrated pneumotachometer and analyzed a representative mean bin (2‐min), this hyperventilation effect may have been due in part to the known stimulatory effects of a mouthpiece and nose clip on eliciting relative hyperventilation in some participants (e.g., Askanazi et al., 1980; Maxwell et al., 1985; Sackner et al., 1980; Scott, 1993; Weissman et al., 1984), combined with participant inexperience with respiratory instrumentation combined. Second, as we outline in the methods, the portable capnograph we utilized for P_ET_CO_2_ measures has a validated atmospheric pressure range up to ~3200 m. Thus, measures of P_ET_CO_2_ above this altitude may depart incrementally from accuracy with further ascent (see Isakovich et al., n.d.). Indeed, we showed that using this model of capnograph with incremental ascent, the P_ET_CO_2_‐PaCO_2_ difference is exaggerated with ascent to 5160 m (Isakovich et al., n.d.; Zouboules et al., 2018). With the assumption that the P_ET_CO_2_‐PaCO_2_ difference (~1–2 mm Hg; Robbins et al., 1990) is unchanged while breathing ambient air at rest with ascent (e.g., Ito et al., 2008), we corrected for the exaggerated underestimation of our P_ET_CO_2_ values with ascent using a linear regression model from a large sample of within‐individual PaCO_2_ and P_ET_CO_2_ values obtained during an identical ascent profile (Zouboules et al., 2018); see Methods and (Isakovich et al., n.d.). These issues with measures of ventilation and P_ET_CO_2_ (i.e., capnography) underscore the importance of utilizing measurement devices that can be both portable and accurate in HA fieldwork contexts when aiming to assess chemoreflex function and VA.

An additional consideration is that our SSCD index only takes into account the prevailing CO_2_, but rapid ascent to HA also imposes an acid–base dysregulation (e.g., respiratory alkalosis), potentially affecting chemoreflex drive independent of CO_2_ (e.g., Fan et al., 2010; Forster et al., 1975; Powell, 2007). However, a previous study from our group showed that arterial pH was fully compensated following one night's sleep at 4240 m following incremental ascent (Zouboules et al., 2018), suggesting that renal compensation was likely complete in our NAz group, leaving the only relevant acid–base variable the metabolic acidosis imposed by the oral acetazolamide administration in the Az subgroup. However, caution should be employed under conditions of more rapid and/or higher ascent profiles, given that above a threshold altitude, it appears that participants are unable to fully compensate from an acid–base perspective (c.f., Forster et al., 1975; Steele et al., 2022 at 4300 m vs. Bird, Leacy, et al., 2021 at 3800 m), and thus all tests of respiratory chemoreflex function may be subject to this caveat. Regardless, in conjunction with the slower ascent profile to 4240 m over 7 days, it is unlikely that participants in either group experienced acid–base dysregulation from an altitude‐mediated hypocapnia/respiratory alkalosis perspective. Accordingly, any acid–base‐related respiratory stimulation likely resulted from the prophylactic dose of acetazolamide in the Az subgroup, explaining the increases in ventilation and SSCD at 4240 m in the Az subgroup compared to the NAz subgroup.

Lastly, in developing the SSCD index, our aim was to assess chemoreflex drive in the steady‐state, without utilizing complex and confounded transient gas tests that lack feasibility in high‐altitude fieldwork contexts. However, one potential critique of the SSCD metric is that it indexes ventilation against a chemostimuli term, where the stimulus index (P_ET_CO_2_/SpO_2_) assumes a 1/1 contribution of these chemostimuli to ventilatory drive. Laboratory studies in humans and reduced animal model preparations suggest that contributions from CO_2_ and O_2_ acting on central and peripheral chemoreceptors change depending upon the activation state and responsiveness of chemoreceptors. For example, the acute HVR test captures peripheral chemoreflex sensitivity, but in the poikilocapnic condition, which is most relevant in the context in HA ascent, the ventilatory response to hypoxia reduces CO_2_, blunting both peripheral and central chemoreceptor contributions to subsequent ventilatory drive. Accordingly, in acute poikilocapnic hypoxia, ventilation after 20‐min of hypoxia is not statistically higher than baseline (Steinback & Poulin, 2007). With VA, the peripheral chemoreflex increases its sensitivity to hypoxia at the prevailing CO_2_ (Duffin & Mahamed, 2003; Loeschcke & Gertz, 1958; Teppema & Dahan, 2010). However, with ascent, participants are chronically hypocapnic, and variability in renally mediated bicarbonate elimination renders acid–base conditions different from baseline at sea level (e.g., Bird, Leacy, et al., 2021; Zouboules et al., 2018). Thus, in low versus high altitude contexts, the challenges of (a) differential background CO_2_/[H^+^] and brain and blood buffering capacity (e.g., HCO_3_^−^), (b) the known CO_2_‐O_2_ chemostimuli interaction at the carotid body (e.g., Lahiri & DeLaney, 1975) and (c) the potential for central‐peripheral chemoreceptor interaction (e.g., Wilson & Teppema, 2016) make the issue of assessing relative chemostimuli and contributions of chemoreceptor compartments in humans with acclimatization to HA nearly impossible. These methodological caveats represent the complexity that we aimed to overcome in the development of the SSCD. We suggest that the considerations of separating contributions from chemostimuli and chemoreceptor compartments are less important in the integrated system when the feedback loops are intact and participants are breathing ambient air in the steady‐state. Assessing the steady‐state ventilatory strategy employed in response to prevailing chemostimuli is likely more representative and important to assess than quantifying a single transient peak response to a transient gas challenge (e.g., Bruce et al., 2018; Steinback & Poulin, 2007).

## 5 CONCLUSIONS

This study is the first to characterize a novel index of steady‐state chemoreflex drive (SSCD) in the context of incremental ascent to high altitude (HA) in two groups of trekkers: one acetazolamide‐free (NAz) and one taking an oral prophylactic dose (125 mg BID) of acetazolamide (Az) during ascent. We conclude that the SSCD metric captures VA during incremental HA ascent, with the associated measures being simple, safe, portable, and feasible in HA fieldwork contexts. Specifically, ventilation and SSCD were larger following incremental ascent, suggesting SSCD captures VA over a time course of 7 days following incremental ascent to 4240 m, representing the steady‐state ventilatory strategy utilized by individuals chronically exposed to hypobaric hypoxia. Additionally, our data demonstrate that VA magnitude is augmented in a group taking a prophylactic dose of oral Az compared to NAz during incremental ascent. However, self‐reported AMS scores were not related to the degree of VA assessed via increases in resting ventilation or SSCD, likely due to the low AMS scores associated with incremental ascent. We suggest that the SSCD index holds promise as a simple, portable, and reliable tool reflecting an integrated assessment of prevailing central and peripheral chemoreflex drive associated with exposure to HA. We encourage other research groups to employ the SSCD metric for studies related to chemoreflex control of breathing to further characterize its utility across a variety of ascent profiles (incremental vs. rapid ascent and residence), duration and with/without prophylactic or treatment doses of acetazolamide.

## AUTHOR CONTRIBUTIONS

Conception and design of the work: KDO, TDB, MTS, TAD; Acquisition, analysis, or interpretation of data for the work: All co‐authors; Drafting the work or revising it critically for important intellectual content: All co‐authors. In addition, all co‐authors, approved the final version of the manuscript, agree to be accountable for all aspects of the work in ensuring that questions related to the accuracy or integrity of any part of the work are appropriately investigated and resolved, all persons designated as authors qualify for authorship, and all those who qualify for authorship are listed.

## FUNDING INFORMATION

Financial support for this work was provided by (a) Alberta Government Student Temporary Employment Program, (b) Alberta Innovates Health Solutions Summer Studentships, (c) Natural Sciences and Engineering Research Council (NSERC) Undergraduate Student Research Assistantships, (d) and NSERC Discovery Grant Program (TAD; PGPIN‐2016‐04915). JKL is funded by the Department of Physiology, University College Cork, Ireland.

## CONFLICT OF INTEREST

None declared.

## ETHICS STATEMENT

This study abided by the Canadian Government Tri‐Council policy on research ethics with human participants (TCPS2) and the Declaration of Helsinki, except for registration in a database. Ethical approval was received in advance through Mount Royal University Human Research Ethics Board (Protocols 2015‐26b and 100012) and was harmonized with the Nepal Health Research Council (Protocols 96–2015 and 109–2017).
